# Desert *Chlorella* Malate Synthase 1 Enhances Salt Tolerance by Promoting Soluble Sugar and Lipid Accumulation

**DOI:** 10.3390/plants15111655

**Published:** 2026-05-28

**Authors:** Yongshun Zhou, Ruyue Tan, Kai Han, Lu Wang, Kaile Fan, Min Chen, Jianfeng Gao, Fulong Chen, Lei Wang

**Affiliations:** 1 Key Laboratory of Xinjiang Phytomedicine Resource and Utilization of Ministry of Education, College of Life Sciences, Shihezi University, Shihezi 832000, China; zhouyongshunyy@shzu.edu.cn (Y.Z.); tanruyue00@163.com (R.T.); 18353169913@163.com (K.H.); wanglu123@stu.shzu.edu.cn (L.W.); a109815886@126.com (K.F.); 2 Xinjiang Production and Construction Corps Key Laboratory of Oasis Town and Mountain-Basin System Ecology, College of Life Sciences, Shihezi University, Shihezi 832000, China; 3 Institute of Information Science and Technology (Institute of Cyber Security), Shihezi University, Shihezi 832000, China; cm_inf@shzu.edu.cn

**Keywords:** desert *Chlorella*, salt stress, malate synthase 1, soluble sugar, lipid metabolism

## Abstract

Desert microalgae possess a remarkable tolerance to extreme abiotic stresses; however, the molecular mechanism underlying their stress adaptation and metabolite accumulation remains poorly understood. In the present study, desert *Chlorella* sp. TLD 6B was exposed to different concentrations of NaCl to assess changes in the soluble sugar and lipid content, as well as transcriptome profiles. The regulatory role of malate synthase 1 (*MS1*) in modulating soluble sugar and lipid accumulation was further investigated in desert *Chlorella* under salinity conditions. The results revealed that salt stress markedly elevated soluble sugar and lipid levels in desert *Chlorella*, while strongly upregulating the transcript expression and enzymatic activity of MS1. Under salt stress, the overexpression of *MS1* in desert *Chlorella* increased the soluble sugar content by 46.49% and the lipid content by 43.87%. The ectopic overexpression of desert *Chlorella MS1* in *Arabidopsis* enhanced salt and drought tolerance without impacting normal plant growth. Mechanistically, MS1 activation modulates metabolic fluxes of the glyoxylate cycle (GAC) and TCA, thereby promoting soluble sugar and lipid accumulation in *Chlorella*. These findings advance the understanding of GAC-governed sugar and lipid accumulation in desert *Chlorella* under salt stress, laying a theoretical foundation for the exploitation of desert microalgae resources in biomass energy development.

## 1. Introduction

Algae are widely distributed in freshwater, seawater, humid soil, deserts, and some extreme environments [1]. Xinjiang harbors two major desert regions, where biological soil crusts host abundant microalgae resources. Desert microalgae exhibit an extraordinary tolerance to extreme environments, such as drought, salinity, and high temperatures, and have evolved unique adaptive strategies to harsh environments [2,3]. Nevertheless, the molecular mechanisms underlying their stress responses remain poorly explored. Microalgae biomass (carbohydrates, lipids) can be converted into clean biofuels such as biodiesel, bioethanol, butanol, and biohydrogen [4,5,6]; therefore, microalgae are regarded as ideal feedstocks for the third generation of biomass energy production [7,8]. Compared with terrestrial crops, microalgae feature a faster growth rate, do not occupy agricultural land, and can be cultivated using wastewater, which will not cause problems such as food security. In contrast to lignocellulose, microalgae possess a simple cellular structure, which is easy to handle. Moreover, they can assimilate CO_2_ via their own photosynthesis, helping mitigate rising carbon emissions. Collectively, microalgae are recognized as one of the most promising, cost-effective, and most economically sustainable raw materials for clean energy supply [7,9,10,11]. In addition, Xinjiang possesses abundant desert microalgal resources, representing a great potential for natural cultivation and novel stress-tolerant gene mining. The vast desert landscapes in Xinjiang also provide favorable conditions for the large-scale exploitation and utilization of microalgae resources. The development of microalgae bioenergy in desertification areas can effectively alleviate the energy crisis and environmental pressures in China and can further contribute to achieving the national double carbon goal.

The carbohydrate content and composition of algae are different. In microalgae, carbon assimilation and carbohydrate biosynthesis primarily occur in chloroplasts via the Calvin cycle [12,13]. Microalgae synthesize starch as the main energy-storing polysaccharide, while cyanobacteria accumulate glycogen. The starch content in microalgae is obviously modulated by the growth environment [14]. Starch and cellulose, located in plastids and cell walls respectively, can be converted into fermentable sugars. Owing to the absence of lignin in microalgae, these carbohydrates can be efficiently hydrolyzed to quickly release sugars, thereby highlighting the great potential of carbohydrate-rich microalgae as promising feedstocks for biofuel production [15,16]. During photosynthesis, microalgae fix carbon dioxide through the Calvin cycle to synthesize glucose, which is subsequently converted into pyruvate by glycolysis. Pyruvate is then catalyzed by the pyruvate dehydrogenase complex to generate acetyl-CoA, which enters the fatty acid synthesis pathway as a substrate of acetyl-CoA carboxylase to produce saturated C16 or C18 fatty acids [17]. Under abiotic stress conditions, microalgae will rapidly degrade their photosynthetic membranes and accumulate triacylglycerol (TAG)-rich liposomes [18]. Microalgae exhibit a broader fatty acid distribution than plants. In addition to the main C16 and C18 fatty acids, some microalgae contain a large amount of C12–C14 and C20–C22 fatty acids, as well as highly unsaturated fatty acids with 3–6 double bonds, which substantially determine their properties as biodiesel [19]. Moreover, microalgae biodiesel displays a superior performance in cold filter plugging points, density, kinematic viscosity, and oxidation stability than paraffin, naphthene, and aromatic hydrocarbon mixtures in diesel [20].

Isocitrate lyase (ICL) and malate synthase (MS) are key enzymes in the glyoxylate cycle, which convert isocitrate into glyoxylate and succinic acid by bypassing the decarboxylation step of the tricarboxylic acid cycle. This prevents carbon loss and provides a crucial carbon skeleton for the synthesis of phospholipids and sterol lipids. In *Candida lipolytica* under the condition of oleic acid as the carbon source, over 80% of the acetyl coenzyme A produced undergoes the GAC pathway metabolism [21]. This pathway releases less carbon and has a higher carbon utilization rate compared to the tricarboxylic acid cycle. In the acetyl-CoA cycle, the malate dehydrogenase catalytic step can generate NADH, which provides reducing equivalents for fatty acid synthesis. When the GAC of *Pseudomonas aeruginosa* is blocked, it will lead to the collapse of the energy metabolism system, with significant decreases in ATP, NAD, and NADP levels [22]. Some microorganisms upregulate the expression of GAC key enzyme genes under high salt levels [23], low temperatures [24,25], drought [26], oxidative stress, antibiotic stress, and host infection [27,28,29,30], which indicates that the glyoxylate cycle plays an important role in stress defense and pathogenesis. In addition, the high-oil-producing strain WJ11 [31] and the MT-overexpressing *M. circinelloides* [32] are endowed with higher glyoxylate cycle fluxes, providing more NADPH and substrate CoA for fatty acid synthesis. The key enzyme genes of the glyoxylate cycle have been confirmed in some microalgae [33,34]. *Chlamydomonas reinhardtii* ICL1 mutants fail to utilize acetate, likely owing to the functional impairment of the glyoxylate cycle [35]. Under high temperature stress, the glyoxylate cycle enzyme gene was differentially expressed in *Symbiodinium* sp. [36]. In previous studies, we found that the key enzyme genes of the glyoxylate cycle in desert *Chlorella* were significantly upregulated under NaCl and PEG stress [37]. However, the function and regulation mechanism of the glyoxylate cycle in the stress resistance and lipid and soluble sugar accumulation of microalgae are still unclear and need to be further investigated.

Desert *Chlorella* rapidly regulates soluble sugar/lipid metabolism under environmental stress, and its glyoxylate cycle is also involved in the regulation of soluble sugar and lipid accumulation. But the regulation mode is poorly understood. Using genetic engineering approaches, we elucidated the regulatory role of the key glyoxylate cycle gene *MS1* in modulating soluble sugar/lipid accumulation in microalgae, providing a theoretical reference for understanding the molecular mechanism of carbon allocation and metabolite accumulation in microalgae. Meanwhile, this establishes a solid theoretical basis for the exploitation and application of Xinjiang desert microalgae as a promising biomass energy resource, thereby helping to alleviate the energy shortage, effectively decrease carbon emissions, and promote the healthy and sustainable development of the microalgae industry.

## 2. Results

### 2.1. Analysis of Changes in Glucose, Lipids, and Transcriptional Profiles of Desert Chlorella Under Different Concentrations of Salt Stress Conditions

**Figure 1:**
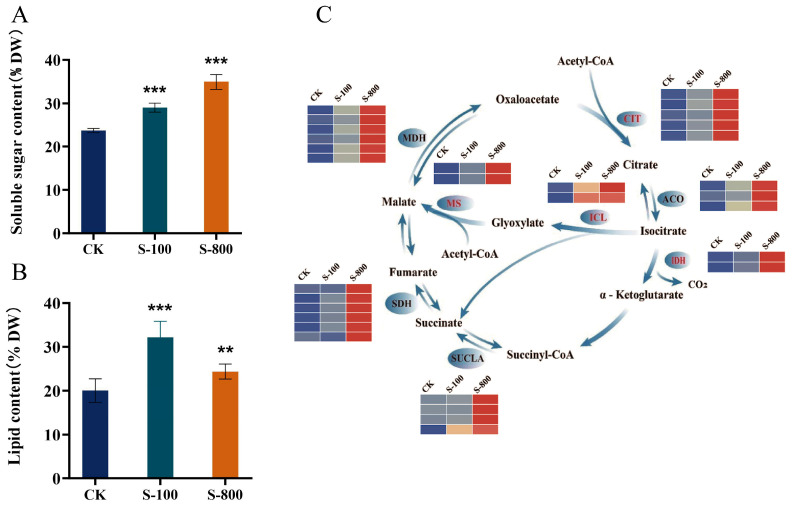
Changes in sugars, lipids, and gene expression profiles of the desert *Chlorella* under different concentrations of salt stress. (**A**,**B**) Changes in soluble sugars and lipids of desert *Chlorella* during salt stress. (**C**) Heatmap showing the changes in transcriptional levels of genes related to the TCA and the GAC in desert *Chlorella* under salt stress. The data represent the average values of three independent biological samples. The asterisks indicate a significant difference at the *p* < 0.05 level, which was determined through Duncan’s multiple range test (**: *p* < 0.01, ***: *p* < 0.001).

To elucidate the correlation between soluble sugar and lipid metabolism and the salt stress response, we determined the contents of these substances in desert *Chlorella* under a NaCl treatment. The results showed that the soluble sugar content increased significantly by 22.2% under 0.1 M NaCl and rose by 47.3% under severe salt stress of 0.8 M NaCl (Figure 1A). Salinity treatments markedly altered the total lipid accumulation in desert *Chlorella*; the lipid content increased by 60.8% under 0.1 M NaCl stress, in contrast to a 21.7% elevation under severe salt conditions (Figure 1B). A heat map of genes involved in the tricarboxylic acid cycle (TCA) was generated according to our previous transcriptome analysis [37] (Figure 1C). The expression levels of the citrate synthase, aconitase, isocitrate dehydrogenase, succinyl-CoA synthase, succinate dehydrogenase, and malate dehydrogenase were all significantly upregulated under salinity stress conditions (Figure 1C). In addition, the key glyoxylate cycle (GAC) enzymes isocitrate lyase and malate synthase were also markedly induced under the salt treatment relative to the control group.

### 2.2. Changes in MS Transcript Level and Enzymatic Activity in Desert Chlorella Under Different Concentrations of NaCl Stress

**Figure 2:**
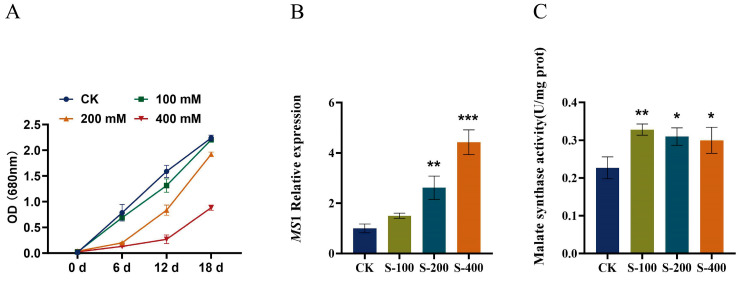
The growth, malate synthase gene expression and enzyme activity of desert *Chlorella* under NaCl stress. (**A**) The growth of desert *Chlorella* under different concentrations of NaCl stress. (**B**) Changes in the transcript level of the *MS1* gene under different concentrations of NaCl treatments. (**C**) Changes in the MS enzyme activity under different concentrations of NaCl treatments. CK: No NaCl; S-100: 0.1 M NaCl; S-200: 0.2 M NaCl; and S-400: 0.4 M NaCl. The data represent the average values of three independent biological samples. The asterisks indicate a significant difference at the *p* < 0.05 level, which was determined through Duncan’s multiple range test (*: *p* < 0.05, **: *p* < 0.01, ***: *p* < 0.001).

Elevated NaCl levels from 100 to 400 mM suppressed the desert *Chlorella* growth in a dose-dependent fashion, with stronger inhibition observed at higher concentrations (Figure 2A). Following 6 days of exposure to different NaCl concentrations, the transcript level of *MS1* was determined (Figure 2B). *MS1* was significantly upregulated under the 0.2 and 0.4 M NaCl stress, reaching 2.61-fold and 4.42-fold higher levels relative to the control, respectively. These results indicate that the glyoxylate cycle is involved in the stress tolerance response of desert *Chlorella*. The key enzymatic activities of the key GAC components were further tested under different concentrations of NaCl treatments (Figure 2C). The activity of the MS enzyme significantly increased under 0.1, 0.2, and 0.4 M NaCl, with 1.45-fold, 1.37-fold, and 1.32-fold increases compared with the control group, respectively, indicating that the MS enzymatic activity responded to the NaCl stress.

### 2.3. An Evolutionary Analysis and Subcellular Localization of MS1 in Desert Chlorella

**Figure 3:**
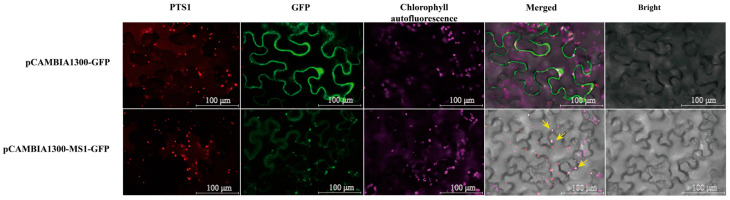
Subcellular localization map of MS1 protein of desert *Chlorella* in tobacco (10 × 20). pCAMBIA1300–GFP and pCAMBIA1300-MS1-GFP fusion proteins were transiently expressed in *N. tabacum* leaves. The fields included mCherry fluorescence of the peroxisome marker (560 nm), green fluorescence field (488 nm), chloroplast autofluorescence field (640 nm), bright field, and merged filed. Empty vector control showing the expression of GFP in epidermal cells of *N. tabacum* leaves and co-localization of GFP, PTS1, and MS1 proteins observed by chloroplast autofluorescence. The yellow arrows indicate the overlap of GFP fluorescence and the autofluorescence of chloroplasts. Bars = 100 μm.

The MS1 fusion expression vector and peroxisome marker (pCAMBIA1300-mcherry: PTS1, purchased from MiaoLingBio, Wuhan, China) were simultaneously transiently transformed into tobacco by the *Agrobacterium* GV3101-mediated method and observed under a laser confocal microscope (Figure 3). pCAMBIA1300 was distributed throughout the tobacco cells, and pCAMBIA1300-mcherry: PTS1 was dispersed in a dot-like manner in the cytoplasm. The green fluorescence of pCAMBIA1300 was overlapped with the red fluorescence of pCAMBIA1300-mcherry: PTS1. The chloroplast autofluorescence did not overlap with the empty vector and peroxisome marker. The result showed that the green fluorescence of MS1 overlaps with the autofluorescence of chloroplasts but does not overlap with the peroxisome marker, indicating that MS1 is located in chloroplasts.

**Figure 4:**
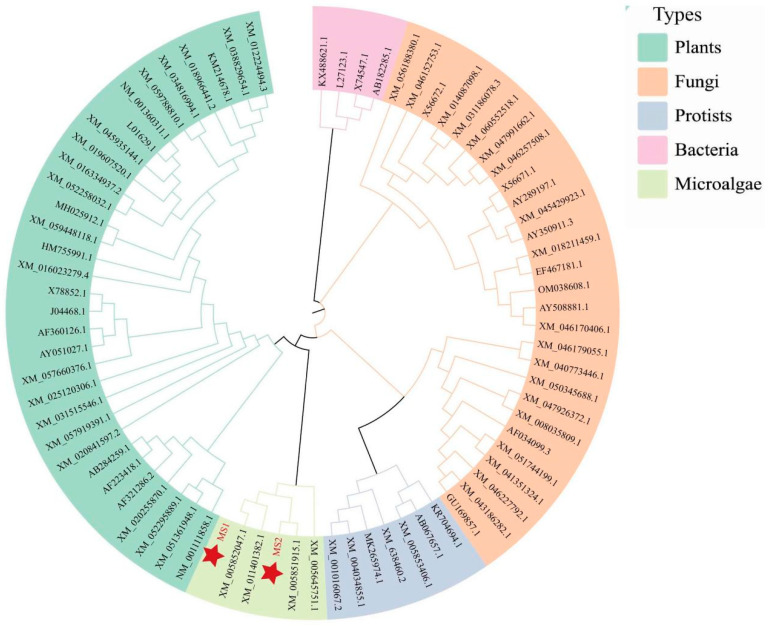
The evolutionary tree of *MS* in desert *Chlorella*. The phylogenetic tree was constructed using the Neighbor Joining method with MEGA 7.0, employing 1000 bootstrap replicates. Homologous *MS* was retrieved from plants, fungi, protists, bacteria and microalgae for phylogenetic comparison. The red star indicates the *MS* of the desert *Chlorella*.

To further explore the phylogenetic relationships of desert *Chlorella MS1* with its homologs in other organisms, the Neighbor Joining method in MEGA 7.0 software was used to analyze the evolutionary relationship. Homologous *MS1* from microalgae, bacteria, prokaryotes, fungi, and plants was included for an evolutionary comparison. A phylogenetic analysis showed that the desert *Chlorella MS1* clustered together with microalgae and plants, indicating the closest genetic affinity to plants. This was followed by a moderate evolutionary relation to prokaryotes and fungi, while exhibiting the farthest phylogenetic distance from bacteria. Collectively, *MS* shares the most close evolutionary relationship with plant homologs (Figure 4).

### 2.4. Changes in Soluble Sugar and Lipid Accumulation in MS1 Transgenic Desert Chlorella Under NaCl Stress

**Figure 5:**
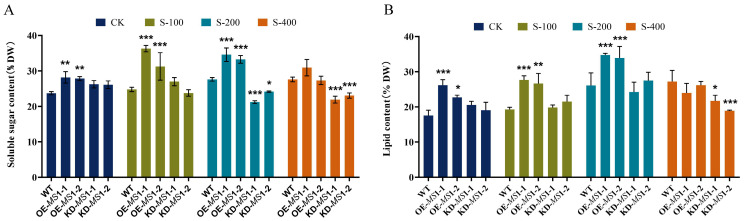
Changes in soluble sugar and lipid contents of *MS1* transgenic *Chlamydomonas* in NaCl stress. (**A**) The changes in soluble sugar content of *MS1*-overexpressing and RNAi desert *Chlorella* during salt stress conditions. (**B**) Changes in lipid content of *MS1*-overexpressing and RNAi desert *Chlorella* under salt stress conditions. WT: Wild-type desert *Chlorella*; OE-MS1: Overexpressed *MS1* desert *Chlorella*; R-MS1: *MS1* RNAi interference desert *Chlorella*; CK: Unstressed; S-100: 0.1 M NaCl stress; S-200: 0.2 M NaCl; S-400: 0.4 M NaCl. The data represent the average values of three independent biological samples. The asterisks indicate a significant difference at the *p* < 0.05 level, which was determined through Duncan’s multiple range test (*: *p* < 0.05, **: *p* < 0.01, ***: *p* < 0.001).

To further characterize the functional role of *MS1* in the salt stress response, we generated *MS1*-overexpression (OE) and *MS1*-RNAi interfering lines of desert *Chlorella*, with wild-type desert *Chlorella* serving as the control for the analysis. All strains were subjected to identical NaCl treatments, and changes in soluble sugar and lipid accumulation were determined under different salinity conditions. Without a NaCl treatment, the soluble sugar content increased significantly in both *MS1*-overexpressing desert *Chlorella* lines, while no significant alteration was observed in the two RNAi lines (Figure 5A). Under 0.1 M NaCl, the soluble sugar content further increased in the both MS1-overexpressing desert *Chlorella* lines, while no significant alteration was observed in the two RNAi lines, as compared to the WT (Figure 5A). The 0.2 M NaCl treatment significantly elevated the soluble sugar levels in the WT, relative to the untreated group. Under 0.2 M NaCl, while both MS1-overexpressing desert *Chlorella* lines still exhibited higher soluble sugar levels than the WT, in contrast, both RNAi lines decreased significantly compared to the WT. Under 0.4 M NaCl, the soluble sugar content decreased significantly in both *MS1*-RNAi lines, further confirming that MS1 plays a crucial role in sugar accumulation in the salt stress response (Figure 5A). At 0.4 M NaCl, soluble sugar levels did not differ significantly between *MS1* overexpression lines and WTs, possibly due to the salt-stress-induced activation of *MS1* in the wild type. Overexpressing lines displayed consistent and significant increases in soluble sugar contents under 0.1 M and 0.2 M NaCl, with maximum elevation rates of 31.7% and 20.0%, respectively (Figure 5A). Therefore, desert *Chlorella MS1* overexpression can stably enhance soluble sugar accumulation under moderate salt stress (0.1 M and 0.2 M NaCl).

Similarly, without the NaCl treatment, the lipid content increased significantly in both *MS1*-overexpressing desert *Chlorella* strains, while no significant alteration was observed in the two RNAi strains (Figure 5B). With the NaCl treatment, the lipid content increased significantly in the WT, *MS1*-overexpressing strains, and RNAi strains, compared to untreated strains (Figure 5B). Under 0.1 M and 0.2 M NaCl, the lipid content increased significantly in *MS1*-overexpressing desert *Chlorella* strains, while no significant alteration was observed in the two RNAi strains, compared to the WT (Figure 5B). Under 0.4 M NaCl, there was no significant change in the lipid content in both *MS1*-overexpressing desert *Chlorella*, while the lipid content in the two RNAi strains was significantly reduced (Figure 5B). *MS1*-overexpressing strains increased their lipid content significantly by 50.6% and 30.1% under the 0.1 M and 0.2 M NaCl stress, respectively. Therefore, we found that the overexpression of *MS1* in desert *Chlorella* can stably increase the content of soluble sugars and lipids under 0.1 M and 0.2 M NaCl stress. Under high salt stress, the lipid content decreased significantly in both *MS1*-RNAi lines, further confirming that *MS1* plays a crucial role in lipid accumulation in the salt stress response.

### 2.5. Overexpression of MS1 in Arabidopsis Enhances the Salt and Drought Tolerance

**Figure 6:**
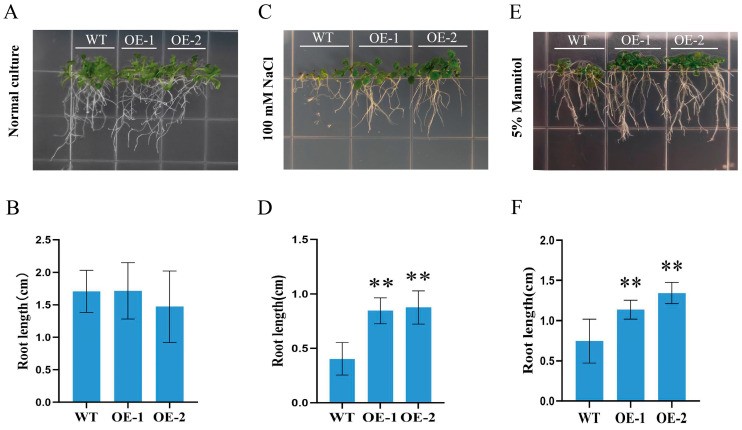
Changes in soluble sugar and lipid contents of *MS1* transgenic *Arabidopsis* in NaCl stress. (**A**) Images of WT and *MS1* transgenic *Arabidopsis* under normal cultivation conditions. (**B**) The statistics of root lengths of wild-type (WT) and MS1 transgenic *Arabidopsis* under normal cultivation conditions. (**C**) Images of WT and *MS1* transgenic *Arabidopsis* under 100 mM NaCl stress. (**D**) The statistics of root lengths of wild-type (WT) and MS1 transgenic *Arabidopsis* under 100 mM NaCl stress. (**E**) Images of WT and *MS1* transgenic *Arabidopsis* under 5% mannitol stress. (**F**) The statistics of root lengths of wild-type (WT) and MS1 transgenic *Arabidopsis* under 5% mannitol stress condition. The asterisks indicate a significant difference at the *p* < 0.05 level, which was determined through Duncan’s multiple range test (**: *p* < 0.01).

To further validate the stress-tolerant function of the desert *Chlorella MS1*, we generated transgenic *Arabidopsis* plants that overexpress *Chlorella MS1*. Under normal growth conditions, no obvious phenotypic differences were observed between transgenic lines and wild-type (WT) plants (Figure 6A,B). Under the salt treatment (Figure 6C,D) and mimicked drought conditions (Figure 6E,F), the two *MS1*-overexpressing lines exhibited superior root growth performance relative to the wild type. These results demonstrated that the heterologous overexpression of the *Chlorella MS1* improves the salt and drought tolerance in *Arabidopsis*. A plausible mechanism is that *MS1* elevates the GAC flux under stress, thereby promoting soluble sugar and lipid accumulation and ultimately improving *Arabidopsis’* stress tolerance.

## 3. Discussion

Abiotic stresses, including salinity, drought, temperature fluctuations, and nutrient limitations, can induce lipid/carbohydrate accumulation in microalgae. Alterations in the soluble sugar concentration have been widely documented in some salt-tolerant plants; for example, the concentrations of glucose, sucrose, fructose, and galactose were significantly higher in salt-tolerant maize varieties than in salt-sensitive maize [38]. Soluble sugar serves as the key substrate for energy production and also participates in stress signal transduction and the modulation of leaf senescence [39]. In addition, the accumulation of soluble sugars contributes to osmotic regulation, thereby constituting an important salt-adaptive mechanism and alleviating salt-induced physiological damage [40]. For instance, elevating the NaCl concentration from 0% to 2% increased the lipid content of *Chlorococcum* sp. from 10.3% to 29.8% [41]. Under salt stress conditions, carbohydrates and lipids are also significantly accumulated in *Scenedesmus* [42]. These published results are consistent with our present observations, in which the soluble sugar and lipid levels were both significantly elevated in desert *Chlorella* under different concentrations of NaCl stress.

Under strong light stress, *H. pluvialis* significantly increased the contents of astaxanthin, carbohydrates, and fatty acids. Proteomic analysis has revealed that the differentially expressed proteins are mainly enriched in pathways related to photosynthetic metabolism, the glyoxylate cycle, and secondary metabolite biosynthesis. The expression of *MS* and *ICL* in rice is significantly upregulated under salt stress [43,44]. The ectopic expression of *Ricinus* communis malate synthase in *Arabidopsis* improves seed germination under heat and salt stress. In addition, the differential expression of the glyoxylate cycle enzyme genes has been detected in *Symbiodinium* sp. under high-temperature conditions [36]. In this study, we demonstrated that the enzyme activity and gene expression of *MS1* were markedly elevated under gradient NaCl treatments. Collectively, these findings highlight that the glyoxylate cycle plays an important role in abiotic stress tolerance, and the glyoxylate cycle likely mediates stress adaptation by modulating the accumulation of soluble sugars and lipids in desert *Chlorella*.

According to the cloned and sequenced *ICL*, *MS1*, and *MS2* genes, a phylogenetic tree was created for analysis. The phylogenetic tree results showed that microalgae *ICL* clustered within the same branch as bacteria homologs and exhibited the farthest evolutionary relationship with plants (Figure S1). This suggests that the *ICL* in desert *Chlorella* may be obtained from bacteria by horizontal gene transfer. By contrast, the *MS1* and *MS2* of desert *Chlorella* grouped into the same branch with plant homologs and were distantly related to prokaryotes and fungi. It is speculated that plant *MS* may have originated from algae through horizontal gene transfer [45]. Different glyoxylate cycle genes within the same species have different evolutionary patterns, revealing that the glyoxylate cycle in desert microalgae has undergone divergent evolution in the face of environmental selection pressure. Such evolutionary divergence facilitates their survival and reproduction in complex and fluctuating environments and also underlies the essence of microalgae biodiversity and strong environmental adaptability.

The system of the nuclear transformation of terrestrial plants using *Agrobacterium* is very common and mature, and the system can also use the pCAMBIA vector to transform microalgae. This has been successfully applied to *Chlamydomonas reinhardtii* [46], *H. pluvialis* [47], *Schizochytrium* [48], and *Isochrysis* sp. [49] using *Agrobacterium*. In addition, the PTS signal peptide sequence was fused with green fluorescent protein for subcellular localization in *Chlamydomonas reinhardtii*, and the GFP observed by fluorescence microscopy in transgenic *Chlamydomonas reinhardtii* was localized in microbodies [50,51]. In this experiment, we used PTS1 as a peroxisome maker for subcellular localization in tobacco. The subcellular localization analysis revealed that desert *Chlorella MS1* is targeted to a specific organelle rather than peroxisome. This finding contradicts previous reports that malic acid synthase is located in the peroxisome microbodies and isocitrate lyase resides in the cytoplasm [33,52]. Such inconsistency may be attributed to the different types of microalgae. The distribution and structure of organelles will also have some differences; this difference may reflect functional differentiation between these two proteins in microalgae, resulting in distinct evolutionary trajectories.

In transgenic desert *Chlorella*, both interference and overexpression altered the sugar/lipid content of desert *Chlorella*, indicating that the regulation effect of the GAC on sugar and lipid metabolism is sophisticated. The manipulation of this pathway may trigger intracellular cascade reactions, leading cells in the direction of sugar and lipid synthesis. Further investigation is still required to understand the regulatory mechanism by which the GAC modulates sugar and lipid accumulation in microalgae. Under NaCl stress (0.1, 0.2, and 0.4 M), the soluble sugar contents were markedly elevated in *MS1*-overexpressing desert *Chlorella*. By contrast, the two RNAi lines exhibited significant reductions in soluble sugar levels under NaCl (0.2, 0.4 M) stress, further confirming that *MS1* plays a crucial role in lipid accumulation in the WT *Chlorella* salt stress response.

The lipid content of *MS1*-overexpressing desert *Chlorella* increased significantly under low and medium concentrations of NaCl stress but did not change significantly under high concentrations of NaCl stress. The lipid content of interfering RNAi in desert *Chlorella* decreased significantly with the increase in the NaCl stress concentration. The increase in the GAC flux provides more energy and substrates for sugar and lipid metabolism, which is consistent with the conclusion that a high GAC flux was found in the high-oil-producing strain WJ11 [31] and the MT-overexpressing *M.circinelloides* [32]. The overexpression of *MS1* in desert *Chlorella* can stably increase the content of soluble sugar and lipids under 0.1 M and 0.2 M NaCl stress. Therefore, the overexpression of *MS1* combined with a 200 mM NaCl stress treatment can achieve the best accumulation of carbohydrates and lipids in desert *Chlorella*. This also provides a theoretical basis for the combined screening of high-yield sugar/lipid microalgae by genetic engineering and abiotic stress.

## 4. Materials and Methods

### 4.1. Desert Chlorella Cultivation and Collection

The sampling site of desert *Chlorella* was the Taklimakan Desert in Xinjiang. The geographical coordinates of the position are 37° 36.732′ N, 80° 23.442′ E. Altitude: 1254 m. The desert *Chlorella* that grew to the logarithmic phase was inoculated into the BBM stress medium supplemented with NaCl (0.1, 0.2, and 0.4 M). At 6 days of stress treatment, the desert *Chlorella* solution was collected by centrifugation with a 50 mL centrifuge tube at 7000 rpm for 15 min, then was washed with distilled water 3 times, frozen in liquid nitrogen for 10 min, and stored in a refrigerator at −80 °C for later use.

### 4.2. Detection of Soluble Sugar and Lipid Content of Desert Chlorella Under Different Concentrations of NaCl Stress

The 10 mg of dry algae powder treated with different concentrations of NaCl was weighed, and 20 mL of distilled water was added. The algae solution was ultrasonically broken at a power of 400 W for 3 s, with a gap of 5 s for a total of 99 times. The soluble sugar content was detected by the anthrone sulfuric acid method: 1 mL of the solution was collected and tested, and 5 mL of the anthrone reagent (Macklin, Shanghai, China) was added; the distilled water was used as the blank control, and the absorbance at a 620 nm wavelength was determined by fully oscillating and mixing, boiling in a water bath for 10 min, and then cooling to room temperature. For the detection of total lipid content, phosphovanillin (Macklin, Shanghai, China) reagent was used: 6 mL chloroform–methanol (Macklin, Shanghai, China) (volume ratio 2:1) solution was added to the disrupted algae solution, shaken overnight, and centrifuged at 4000 rpm for 10 min. The lower liquid was carefully pipetted into a centrifuge tube with a pipette, and then 1 mL of 0.9% NaCl solution was added, shaken, mixed for 1 min to ensure that the solution was mixed evenly, and then centrifuged at 4000 r/min for 10 min to further purify the lipid extract. Then the lower solution was transferred to a 10 mL volumetric flask, and the volume was adjusted to 10 mL with chloroform–methanol solution. Then 100 μL of the solution to be measured was collected in the test tube, and the chloroform was completely volatilized by a boiling water bath for 15 min. When the temperature was reduced to room temperature, 250 μL of concentrated sulfuric acid was added, and the boiling water bath was used for 10 min. After the temperature reduced to room temperature, 5 mL of vanillic aldehyde phosphate chromogenic agent was added, mixed with a vortex shaker, and stood for 1 h, and the absorbance was measured at a wavelength of 528 nm.

### 4.3. Enzyme Activity Detection of MS Under Different Concentrations of NaCl Stress

A total of 0.1 g of desert *Chlorella* treated under different stresses for 6 days was weighed and frozen in liquid nitrogen for 15 min. Then, 1 mL of enzyme extract (Solarbio Science & Technology Co., Ltd., Beijing, China) was added to the mortar, and a small amount of quartz sand was added to grind and homogenize on ice. After centrifugation at 12,000 rpm and 4 °C for 10 min, we carefully drew the supernatant as the test solution, placed it on ice for testing, and added the reagent according to the instructions. The absorbance at 412 nm was measured after fully mixing and standing for 5 min.

### 4.4. The Expression Analysis of the MS1 Under Different Stresses

Total RNA extraction kit (Tiangen Biotechnology, Beijing, China) was used for RNA extraction, and cDNA Synthesis SuperMix reverse transcription kit (TransGen Biotechnology, Beijing, China) was used for reverse transcription. The reaction system was (20 μL): Total RNA 1 μL, Anchored Oligo (dT) 1 μL, 2 × TS Reaction Mix, TransScript RT/RI Enzyme Mix 1 μL, gDNA Remover 1 μL, RNase-free Water 14 μL. After mixing, the mixture was bathed in a 42 °C constant-temperature water bath for 30 min. Then the reverse transcriptase was inactivated in an 85 °C thermostatic water bath for 5 s. The specific primers of the *MS1* were designed by Primer Premier 5: MS1-qp1 CTGGCTCCCCGAGACTA, MS1-qp2 CGCCGCACCGCATCATA. And the MS1 gene was detected by qRT-PCR.

### 4.5. Construction of MS1 Overexpression, RNAi Interference, and Fusion Expression Vectors

Primers were designed according to the full-length transcript of *MS1*: PB-MS1S-1: GCGTCCGATAGCAGGTGAGTAA, PB-MS1AS-1: CAAAGCAACAGGGACGATACAG C. Phanta Max Super-Fidelity DNA Polymerase (Vazyme, Nanjing, China) was used for PCR. The PCR products were recovered according to the gel recovery instructions and transformed into competent cells for sequencing. The *MS1* of desert *Chlorella* was ligated to the plant binary expression vector pCAMBIA1300-GFP by homologous recombination. The pCAMBIA1300 vector was digested with BamH1 and Spe1 (TransGen Biotech, Beijing, China), and the enzyme system was carried out according to the instructions. The overexpressed recombinant plasmid pCAMBIA1300-MS1-GFP was obtained by the ClonExpressII rapid recombination cloning technique. Using the same method, the *MS1* gene of desert *Chlorella* was ligated to the vector pCAMBIA1300-GFP to generate a subcellular localization recombinant plasmid pCAMBIA1300-MS1: GFP. The RNAi interference fragment was designed using the BLOCK-iT RNAi Designer online tool (Thermo Fisher Scientific, Waltham, MA, USA) (accessed 1 March 2022). The *MS1* cloning vector was amplified by PCR with high-fidelity enzyme and specific primers, and fragment 1 (forward repeat sequence) and fragment 2 (reverse repeat sequence) were obtained, respectively, which were named MS1: 1 and MS1: 2, respectively. The MS1: 1 and MS1: 2 fragments were recombined with the pTCK303 interference vector using the above method and named pTCK303-MS1. The above vectors were transformed into competent cells and sequenced.

### 4.6. Agrobacterium-Mediated Transformation of Desert Chlorella and Arabidopsis

*Agrobacterium*-mediated genetic transformation: Desert *Chlorella* was cultured at 25 °C for 6 days until OD680 was about 0.8. Then 1 mL of algae liquid was collected on an ultra-clean bench at 7000 rpm and centrifuged for 5 min. The supernatant was dropped, and sterile water was diluted to OD680 of about 0.1 for use. The successfully transformed overexpressing and RNAi interference *Agrobacterium* GV3101 were streaked and activated and cultured in 50 mL LB medium containing antibiotics to an OD600 of about 0.8. A total of 100 μL of bacterial solution was mixed with 100 μL of desert *Chlorella* solution and inoculated in a common medium. The mixture was cultured in a constant-temperature incubator at 28 °C in the dark for 48 h, and then the co-culture solution was spread on the screening medium at 28 °C for 6 days. The positive transformants were selected and inoculated in BBM medium containing 400 μg/mL cephalosporin and 40 μg/mL hygromycin to expand the culture. The culture was cultured to the logarithmic phase, the algae solution was collected, and DNA was extracted for PCR verification.

### 4.7. Subcellular Localization

*Nicotiana benthamiana* was planted in nutrient-rich soil (the ratio of soil, vermiculite, and perlite was 3:1:1), and the seedlings could be infected when they grew to 45 days. The *Agrobacterium* containing the pCAMBIA1300-MS1: GFP vector was cultured overnight, at 4000 r/min for 20 min, and the bacterial solution was collected by centrifugation at room temperature. The supernatant was discarded, 5 mL of solution was added, the bacteria were washed and centrifuged again at 4000 r/min, and the bacteria were collected for 20 min. Then 1 mL of solution was added to resuspend the bacteria. The value of OD600 was detected and diluted to 0.3. The diluted bacterial solution was then mixed with the bacterial solution containing the peroxisome maker (pCAMBIA 1300-mcherry: PTS1) in a 1:1 ratio. After standing for 1 h, the bacterial solution was gently injected into the back of the tobacco leaves with a 1 mL syringe and marked with a marker pen. After the injection was completed, the cells were cultured normally for 2 days after 12 h in the dark, and the fluorescence was detected by laser confocal microscopy.

### 4.8. Data Processing

All physiological and biochemical data were expressed as mean ± standard deviation of three replicates. One-way analysis of variance (ANOVA) with significance and chart construction were performed using Graphpad Prism 10.1.2 software.

## 5. Conclusions

In summary, by assessing changes in the soluble sugar and lipid content, as well as transcriptome profiles, when exposed to NaCl treatments, we revealed and demonstrated the specific regulatory role of *MS1* in the salt stress response. The soluble sugar and lipid content of *MS1*-overexpressing desert *Chlorella* increased significantly under 0.1 M and 0.2 M NaCl stress, while the soluble sugar and lipid content of the RNAi interference desert *Chlorella* did not change significantly under 0.1 M and 0.2 M NaCl stress. The overexpression of the desert *Chlorella MS1* improved the drought resistance of *Arabidopsis thaliana*. This proves that strengthening the flux of the GAC can promote the accumulation of soluble sugars and lipids; this study provides a theoretical basis and reference for the molecular mechanism of the soluble sugar and lipid accumulation of microalgae and also lays a theoretical foundation for the study of biomass energy, the carbon sink of desert microalgae, and its application for protecting the natural environment and improving the ecological environment in Xinjiang.

## Data Availability

The original contributions presented in this study are included in the article/Supplementary Materials. Further inquiries can be directed to the corresponding authors.

## Supplementary Materials

The following supporting information can be downloaded at https://www.mdpi.com/article/10.3390/plants15111655/s1, Figure S1: Phylogenetic analysis of *ICL* from the desert Chlorella. The phylogenetic tree was constructed using the Neighbor Joining method with MEGA 7.0, employing 1000 bootstrap replicates. Homologous ICL proteins were retrieved from plants, fungi, protists, bacteria and microalgae for phylogenetic comparison.
