# Supplementary material for: Desert Chlorella Malate Synthase 1 Enhances Salt Tolerance by Promoting Soluble Sugar and Lipid Accumulation
Source: Plants (Basel). 2026 May 28;15(11):1655. doi: 10.3390/plants15111655 (PMC13259352; doi:10.3390/plants15111655)
Supplement: Supplementary file 1 [file plants-15-01655-s001.zip › plants-4330559-supplementary.pdf]

Supplementary Materials

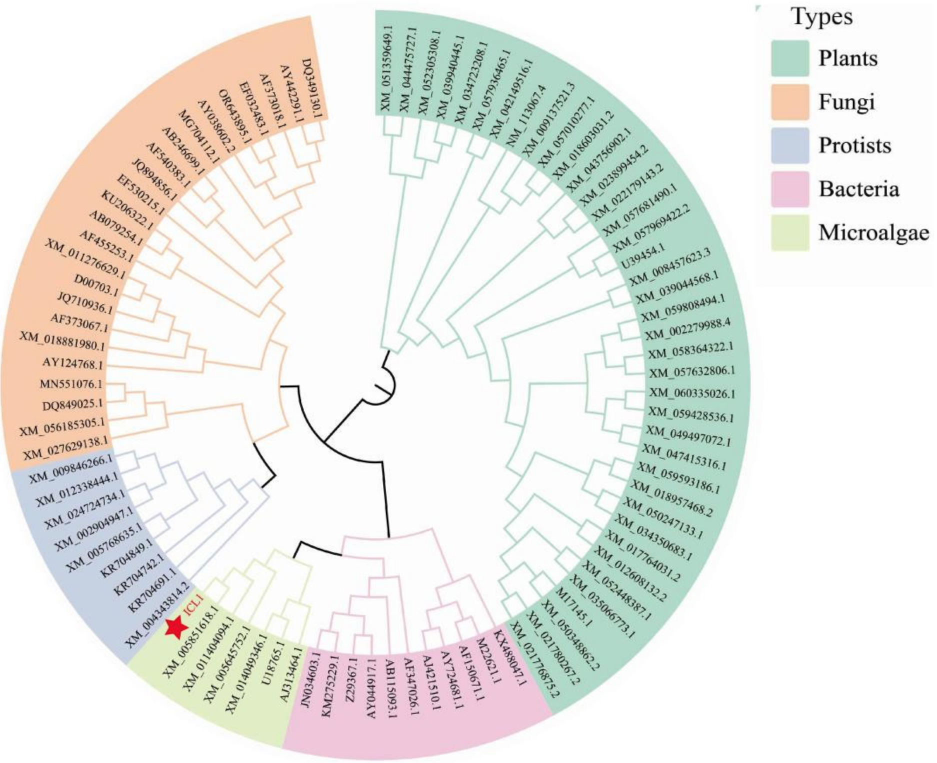

Figure S1. Phylogenetic analysis of *ICL* from the desert Chlorella. The phylogenetic tree was constructed using the Neighbor Joining method with MEGA 7.0, employing 1000 bootstrap replicates. Homologous ICL proteins were retrieved from plants, fungi, protists, bacteria and microalgae for phylogenetic comparison.
